# Direction-of-Arrival Estimation Based on Joint Sparsity

**DOI:** 10.3390/s110909098

**Published:** 2011-09-21

**Authors:** Junhua Wang, Zhitao Huang, Yiyu Zhou

**Affiliations:** School of Electronic Science and Engineering, NUDT, Changsha 410073, China; E-Mails: zhitaohuang@nudt.edu.cn (Z.H.); zhouyiyu@nudt.edu.cn (Y.Z.)

**Keywords:** joint-sparse, compressed sensing, Direction-of-Arrival, quasi-Newton methods, multiple measure vectors

## Abstract

We present a DOA estimation algorithm, called Joint-Sparse DOA to address the problem of Direction-of-Arrival (DOA) estimation using sensor arrays. Firstly, DOA estimation is cast as the joint-sparse recovery problem. Then, norm is approximated by an arctan function to represent joint sparsity and DOA estimation can be obtained by minimizing the approximate norm. Finally, the minimization problem is solved by a quasi-Newton method to estimate DOA. Simulation results show that our algorithm has some advantages over most existing methods: it needs a small number of snapshots to estimate DOA, while the number of sources need not be known *a priori*. Besides, it improves the resolution, and it can also handle the coherent sources well.

## 1. Introduction

Direction-of-Arrival (DOA) estimation using sensor arrays has been an active research area, playing a fundamental role in many applications involving electromagnetic, acoustic, and communication systems [1]. Many classical algorithms are available, and the popular methods include beamforming [2], MUSIC [3], ESPRIT [4] and the maximum likelihood method [5], *etc.* The beamforming method has low angle resolution and suffers from the Rayleigh resolution limit. MUSIC, ESPRIT and the maximum likelihood method all rely on the statistical properties of the data, and thus, require a sufficiently large number of samples for accurate estimation. Besides, MUSIC and ESPRIT cannot handle strongly coherent sources, while the maximum likelihood method has high computation costs.

The problem of sparse recovery has evolved rapidly recently [6,7] and it has been applied in DOA estimation with array processing. Gorodnitsky *et al.* [8] used a weighted least-squares algorithm named FOCUSS for DOA estimation, but this algorithm can only be used for single snapshots. Cotter [9] combined multiple measurement vectors (MMV) and matching pursuit (MP) to solve the joint-sparse recovery problem in DOA estimation, but it has low angle resolution. JLZA-DOA is proposed in [10]; it minimizes a mixed *L*_2,0_ norm to deal with the joint-sparse recovery problem, and a fixed point method is used for DOA estimation. This algorithm doesn’t satisfy numerical stability, as matrix inversion is inevitable in every iteration. Stoica *et al.* [11] presented a novel SParse Iterative Covariance-based Estimation approach, abbreviated SPICE. However, this algorithm needs more snapshots to estimate DOA. Wide-band covariance matrix sparse representation (W-CMSR) is proposed in [12] for DOA estimation of wideband signals. So far, the most successful joint-sparse recovery algorithm for DOA estimation is L1-SVD [13,14]. It combines the SVD step of the subspace algorithms with a sparse recovery method based on *l*_2,1_ –norm minimization. However, the number of sources needs be known *a priori*.

In this paper, we present Joint-Sparse DOA estimation, abbreviated as JSDOA, for sensor array DOA estimation. First, DOA estimation is cast as a joint-sparse recovery problem. Then, *L*_2,0_ norm is approximated by the arctan function to represent spatial sparsity and DOA estimation can be obtained by minimizing the approximate *L*_2,0_ norm. Finally, the minimization problem is solved by a quasi-Newton method to estimate DOA. The proposed algorithm has some advantages over most existing methods: it needs a small number of snapshots to estimate DOA, an the number of sources need not be known *a priori*. Besides, it improves the probability of resolution, and it can also handle coherent sources well.

The outline of the paper is as follow. In Section 2, the DOA estimation problem is formulated. The new algorithm, called JSDOA, is proposed in Section 3. In section 4, the validity of the proposed algorithm is proved by a number of simulations. Finally, conclusions are presented in Section 5.

## 2. Problem Formulation

### 2.1. DOA Estimation Problem

Consider a linear array consisting of *M* identical sensors and receiving signals from *K* narrowband signals *s*_1_(*t*), *s*_2_(*t*), ⋯, *s_K_*(*t*), which arrive at the array from directions *θ̄*_1_, *θ̄*_2_, ⋯ *θ̄_K_* with respect to the line of array. The received signal *y_m_*(*t*) at the *m^th^* sensor can be written as:

(1) $$y_{m}(t)=\sum_{k=1}^{K}a({\bar{\theta }}_{k})s_{k}(t)+n_{m}(t)$$

where ${\left\{a({\bar{\theta }}_{k})\right\}}_{k=1}^{K}$ denote steering vectors, *n_m_*(*t*) (*m* = 1, 2 ⋯, *M*) stand for the additive noise.

Let *y*(*t*) = [*y*_1_(*t*), *y*_2_(*t*), ⋯, *y_M_*(*t*)]*^T^*, *n*(*t*) = [*n*_1_(*t*), ⋯, *n_M_*(*t*)], Equation (1) can be written as:

(2) $$y(t)=A(\bar{\theta })s(t)+n(t)$$

where the manifold matrix *A* (*θ̄*) consists of the steering vectors ${\left\{a({\bar{\theta }}_{k})\right\}}_{k=1}^{K}$:

$$A(\theta )=\left[a({\bar{\theta }}_{1}),a({\bar{\theta }}_{2}),\cdots ,a({\bar{\theta }}_{K})\right]$$

Therefore, DOA estimation is to find *K* and *θ̄_k_* from *T* snapshots ${\left\{y(t)\right\}}_{t=t_{1}}^{t_{T}}$.

### 2.2. Joint-Sparse Recovery for DOA Estimation Problem

Because sources are sparse in space, DOA estimation with sensor arrays can be expressed as a joint-sparse recovery problem. Let Ω denote the set of possible locations, ${\left\{\theta_{n}\right\}}_{n=1}^{N}$ denotes a grid that covers Ω. We assume that the grid is fine enough such that the true location parameters of the existing sources lie on the grid. Let:

$$x(t)={\left[x_{1}(t),\;x_{2}(t),\;\cdots ,x_{N}(t)\right]}^{T}$$

Then the received signal *y_m_*(*t*) at the *m^th^* sensor can be written as:

(3) $$y_{m}(t)=\sum_{n=1}^{N}a(\theta_{n})x_{n}(t)+n(t)$$

where the *n^th^* element *x_n_*(*t*) of *x*(*t*) is nonzero only if *θ_n_* = *θ̄_k_*, *k* ∈ [1,2, ⋯, *K*] and in that case *x_n_*(*t*) = *s_k_*(*t*):

Let Φ = [*a*(*θ*_1_), *a*(*θ*_2_), ⋯, *a*(*θ_N_*)], (3) can be expressed as:

(4) y(t)=Φx(t)+n(t)

when the number of snapshots is denoted as *T*, Equation (4) can be written as:

(5) Y=ΦX+N

where *Y* = [*y*(*t*_1_), *y*(*t*_2_), ⋯, *y*(*t_T_*)], *X* = [*x*(*t*_1_), *x*(*t*_2_), ⋯, *x*(*t_T_*)]. As we know, *X* is row-sparse and only *K* rows have nonzero elements, so DOA estimation can be obtained by a joint-sparse recovery problem, which is also called a multiple measurement vectors (MMV) problem. Using *l_p,q_* norm to express joint sparsity, the MMV problem can be converted to *l_p,q_* norm minimization:

(6) $$\{\begin{array}{ll} \text{min} & {\left\|X\right\|}_{p,q}\\ s.t. & Y=\Phi X+N \end{array}$$

where *p* and *q* are non-negative, and ‖*X*‖*_p,q_* is defined as:

(7) $${\left\|X\right\|}_{p,q}=\sum_{i=1}^{M}{\left({\left\|X(i,:)\right\|}_{p}\right)}^{q}$$

Concretely, Eldar and Mishali [15] use *p =* 2, *q* = 1. Chen and Huo [16] study for *p =* 1, *q* = 1. The above algorithms do not perform very well for ‖*X*‖ _2,1_ and ‖*X*‖ _1,1_ can’t sufficiently reflect joint sparsity. Considering ‖*X*‖ _2,0_ can reflect joint sparity sufficiently, we minimize ‖*X*‖ _2,0_ norm to solve the MMV problem. However, ‖*X*‖ _2,0_ norm minimization can hardly be solved directly. Therefore, in this paper we approximate ‖*X*‖ _2,0_ norm by an arctan function and estimate DOA by solving an approximate ‖*X*‖ _2,0_ norm minimization problem.

## 3. Joint-Sparse DOA Estimation Algorithm

In this section, the DOA estimation problem is converted into an approximate ‖*X*‖ _2,0_ norm minimization problem. Firstly, the *L*_2,0_ norm is approximated by an arctan function to construct an approximate ‖*X*‖ _2,0_ norm minimization problem. Then this problem is solved by a quasi-Newton method to estimate DOA.

### 3.1. Basic Idea of the Proposed Method

Let *ξ_i_* = ‖*X*(*i*,:)‖_2_, *i* = 1, 2, ⋯, *M*, we have:

(8) $${\left\|X\right\|}_{2,0}=\sum_{i=1}^{M}{\left({\left\|X(i,:)\right\|}_{2}\right)}^{0}={\left\|\xi \right\|}_{0}$$

where *ξ* = (*ξ*_1_, ⋯, *ξ_M_*). Considering the following arctan function:

(9) $$f_{\delta }(s)=\frac{2}{\pi }\mathrm{arc}\;\text{tan}(\frac{s^{2}}{2\delta^{2}})$$

where *δ* is a positive parameter and *s* is a variable parameter. Then *f_δ_*(*s*) has the following property:

(10) $$\underset{\delta \to 0}{\text{lim}}f_{\delta }(s)=\{\begin{array}{cc} 1 & s\neq 0\\ 0 & s=0 \end{array}$$

Let

$$F_{\delta }(\xi )=\sum_{i=1}^{N}f_{\delta }(\xi_{i})$$

From Equation (10), we have:

(11) $$\underset{\delta \to 0}{\text{lim}}F_{\delta }(\xi )={\left\|\xi \right\|}_{0}$$

From Equations (8) and (10), then:

$$\underset{\delta \to 0}{\text{lim}}F_{\delta }(X)={\left\|X\right\|}_{2,0}$$

So DOA can be obtained by solving the approximate ‖*X*‖_2,0_ norm minimization:

(12) $$\{\begin{array}{ll} \text{min} & F_{\delta }(X)\\ s.t. & Y=\Phi X+N \end{array}$$

where *δ* is a small positive constant. Because noise is unknown, we synchronously hope to minimize *F_δ_*(*X*) and ${\left\|\Phi X-Y\right\|}_{F}^{2}$. Then Equation (12) is converted into a multiple objective optimization:

(13) $$\{\begin{array}{ll} \underset{X}{\text{min}} & F_{\delta }(X)\\ \underset{X}{\text{min}} & {\left\|\Phi X-Y\right\|}_{F}^{2} \end{array}$$

Using the linear weighting method, Equation (13) can be written as:

(14) $$\underset{x}{\text{min}}\;\;\;L_{\delta ,\lambda }(X)=F_{\delta }(X)+\lambda {\left\|\Phi X-Y\right\|}_{F}^{2}$$

where ‖•‖*_F_* denotes the Frobenious norm and the parameter *λ* will be discussed in Section 3.2. When the parameter *δ* is very small, the objective function *F_δ_*(*X*) is highly unsmoothed and contains a lot of local minimization, so its global minimization is not easy. On the other hand, if the parameter *δ* is larger, the objective function *F_δ_*(*X*) is smoother and contains less local minimization. In order to obtain global minimization, we select a decreasing sequence for *δ*, denoted as:

$$\delta =[\delta_{1},\delta_{2},\cdots ,\delta_{J}],\;\;\delta_{j+1}<\delta_{j}$$

where *δ_1_* is a relatively large value, and *δ_J_* is a small value. For *δ* = *δ_J−1_*, the solution of Equation (14) is denoted as *x*_*δ*_*j*–1__, and *x*_*δ*_*j*–1__ is used as the initial value for *δ* = *δ_j_*, thus we hope the proposed algorithm can escape from getting trapped into a local minimum and reach the global minimization for a small value *δ* = *δ_J_*.

For some fixed value *δ* = *δ_j_*, minimization problem Equation (14) is solved by the quasi-Newton method in this paper. One of the most successful quasi-Newton methods is the BFGS algorithm, which is second order convergent and has good numerical stability. Therefore, we use the BFGS algorithm to solve Equation (14) for some fixed value *δ* = *δ_j_*.

The conjugate gradient for a matrix variable is defined as:

$$\frac{\partial L_{\delta ,\lambda }(X)}{\partial X^{*}}=\frac{1}{2}\left(\frac{\partial L_{\delta ,\lambda }(X)}{\partial X_{R}}+i\frac{\partial L_{\delta ,\lambda }(X)}{\partial X_{I}}\right)$$

where *X_R_* and *X_l_* denote the real part and the imaginary part respectively, X^*^denotes the conjugate of *X*. Then the conjugate gradient of *L_δ,λ_*(*X*) (for its derivation see Appendix I) can be expressed as:

(15) $$\frac{\partial L_{\delta ,\lambda }(X)}{\partial X^{*}}=\frac{1}{2\delta^{2}}\Lambda X-\lambda \Phi^{*}(Y-\Phi X)$$

where

$$\Lambda =\mathrm{diag}\left\{\delta^{2}/\left(\xi_{1}^{4}+4\delta^{4}\right),\;\delta^{2}/\left(\xi_{2}^{4}+4\delta^{4}\right),\cdots ,\delta^{2}/\left(\xi_{n}^{4}+4\delta^{4}\right)\right\}$$

Solving by the BFGS algorithm, the main iterative steps are as follows:

1. Search step length *t_k_*, satisfy: $t_{k}=\underset{t}{\text{min}}\;\;L_{\delta_{j},\lambda }\;\left(x-tH^{k}\nabla L_{\delta_{j},\lambda }(X)\right)$
2. Iteration: $X^{k+1}=X^{k}-t_{k}\;\;H^{k}\frac{\partial L_{\delta ,\lambda }(X^{k})}{\partial X^{*}}$
3. BFGS adjustment:
  (16) $$H^{k+1}=H^{k}+\left(1+\frac{{\left(g^{k}\right)}^{T}H^{k}g^{k}}{{\left(g^{k}\right)}^{T}s^{k}}\right)\frac{s^{k}{\left(s^{k}\right)}^{T}}{{\left(g^{k}\right)}^{T}s^{k}}-\frac{s^{k}{\left(g^{k}\right)}^{T}H^{k}-H^{k}g^{k}{\left(s^{k}\right)}^{T}}{{\left(g^{k}\right)}^{T}s^{k}}$$

where *s^k^* = *X*^*k*+1^ – *X^k^*, $g^{k}=\frac{\partial L_{\delta ,\lambda }(X^{k+1})}{\partial X^{*}}-\frac{\delta L_{\delta ,\lambda }(X^{k})}{\partial X^{*}}$

### 3.2. Algorithm Description

Based on the above idea, JSDOA can be described as shown in Table 1.

Remark:

**(1) Select parameter** *λ*: in this paper, we select the parameter *λ* by the α– method [17]. Set *f*_1_(*X*) = *F_δ_*(*X*), $f_{2}(X)={\left\|\mathrm{AX}-y\right\|}_{F}^{2}$, and minimizing *f*_1_(*X*) and *f*_2_(*X*), we have;

$$f_{i}(X^{\left(i\right)})=\underset{x}{\text{min}}\;\;f_{i}(X)\;i=1,2$$

It is easy to know that *X*^(*i*)^(*i* = 1,2) is 0 and *A^T^*(*AA^T^*)^−1^*Y* respectively. Then we have:

$$f_{\mathrm{ij}}=f_{i}(X^{\left(j\right)})\;i,j=1,2$$

Set *λ* = *λ*_2_/*λ*_1_ and introduce auxiliary parameter *α*, we can have:

$$\{\begin{array}{l} \sum_{i=1}^{2}f_{\mathrm{ij}}\lambda_{i}=\alpha \\ \sum_{i=1}^{2}\lambda_{i}=1 \end{array}$$

Setting the coefficient matrix *f*(*i*,*j*) = *f_ij_*, from the above equations, the solution is:

(17) $$\{\begin{array}{l} {}[\lambda_{1},\lambda_{2}]=\frac{e^{T}f^{-1}}{e^{T}f^{-1}e}\\ \alpha =\frac{1}{e^{T}f^{-1}e} \end{array}$$

where *e* = [1,1]*^T^*, and *λ* = *λ*_2_/*λ*_1_.

**(2) Select parameter** *δ*: In this paper, we set *δ_j_* = *γδ_j_*_–1_, *j* = 2, ⋯, *J*, *γ* ∈ (0.5,1). Let $\tilde{x}={\text{max}}_{i}\left|x_{i}^{0}\right|$, we hope parameter *δ*_1_ satisfies:

$$f_{\delta }(\tilde{x})=\frac{2}{\pi }\mathrm{arc}\;\text{tan}\;(\frac{{\tilde{x}}^{2}}{2\delta_{1}^{2}})\leq \frac{1}{2}\Rightarrow \delta_{1}\geq \frac{\tilde{x}}{\sqrt{2}}$$

In order to save computation cost, we set $\delta_{1}={\text{max}}_{i}\left|x_{i}^{0}\right|/\sqrt{2}$. When *δ_J_* → 0, *F*_*δ*_*J*__(*x*) → ‖*x*‖_0_. However, if *δ_J_* is too small, *F*_*δ*_*J*__(*x*) will be sensitive to noise, so *δ_J_* shouldn’t be too small.

**(3) For coherent sources:** Many of popular methods, such as MUSIC and ESPRIT require the assumption that sources are uncorrelated, because the source covariance matrix remains nonsingular so long as none of these sources are coherent. However, the algorithm proposed in this paper doesn’t need to compute the source covariance matrix, and joint sparsity is used to estimate DOA, so the proposed algorithm can handle highly coherent sources as well.

**(4) Limitation:** The algorithm proposed in this paper suffers from two problems. First, there exist no clear-cut guidelines for the selection of *δ_J_*, especially when knowledge of noise is unknown. Second, the proposed algorithm can’t perform well when the SNR is relatively low.

## 4. Simulation Results

In this section, we present several numerical simulation results to illustrate the performance of the proposed algorithm. First, we compare the spatial spectrum of JSDOA to those of beamforming [2], MUSIC [3], and L1-SVD [13] under various snapshot scenartios. Next, we compare the spatial spectrum with more than two sources. Then, the probability of resolution is compared. Finally, we compare the spatial spectrum for coherent sources. In the following numerical simulations, we consider a uniform linear array consisting of *M* = 8 identical sensors and receiving signals are narrowband signals. The sensors are uniformly placed with a spacing of half a wavelength. The interval for the DOA is Ω = [−90,90], we use a uniform grid ${\left\{\theta_{n}\right\}}_{n=1}^{N}$ to cover Ω with a step of 1°, which means *N* = 181.

### 4.1. Spatial Spectrum Comparison under Various Snapshots

**Figure 1.**
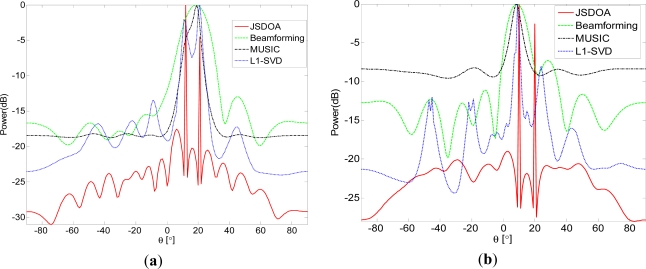
Spatial spectrum comparison under various snapshots for two uncorrelated sources: (**a**) *T* = 10; and (**b**) *T* = 5.

In this simulation, we compare spatial spectrum of different algorithms under various snapshots. We consider two uncorrelated sources located at 13° and 20° with *SNR* = 10. For JSDOA, we set $\delta_{1}={\text{max}}_{i}\left|x_{i}^{0}\right|/\sqrt{2}$, *δ_j_* = *γδ_j_*_–1_, *γ* = 0.5, *J* = 7. In Figures 1(a,b), we set *T* = 10 and *T* = 5, respectively. It can be seen from Figure 1 that JSDOA and L1-SVD can resolve the two sources as *T* = 10. However, only JSDOA can resolve the two sources as *T* = 5.

### 4.2. Spatial Spectrum Comparison with More Than Two Sources

**Figure 2.**
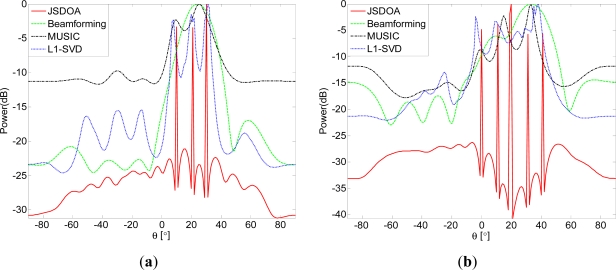
Spatial spectrum comparison for more than two uncorrelated sources: (**a**) Three sources; and (**b**) Five sources.

In this simulation, we compare the spatial spectrum of different algorithms with more than two uncorrelated sources. Set *SNR* = 10, *T* = 20, $\delta_{1}={\text{max}}_{i}\left|x_{i}^{0}\right|/\sqrt{2}$, *δ_j_* = *γδ_j_*_–1_, *γ* = 0.5, *J* = 7. In Figure 2(a), there are three sources located at 10, 20 and 30. In Figure 2(b), there are five sources located at 0, 10, 20, 30 and 40. It is shown from Figure 1 that JSDOA and L1-SVD can resolve the three sources, but only JSDOA can resolve the five sources.

### 4.3. Probability of Resolution Comparison under Various Conditions

**Figure 3.**
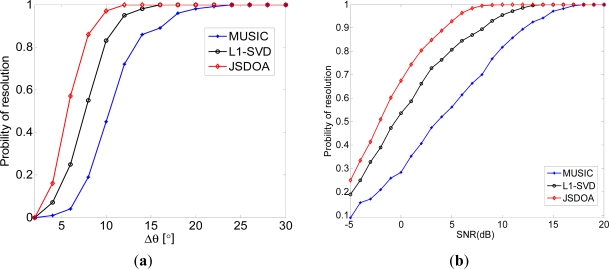
Probability of resolution comparison under various conditions. (**a**) Against Δ*θ*; and (**b**) Against input SNR.

In this simulation, we compare probability of resolution for two uncorrelated sources. JSDOA, MUSIC and L1-SVD are used for comparison and we set *T* = 5, *δ_j_* = *γδ_j_*_–1_, *γ* = 0.5, *J* = 7. For each Δ*θ* or SNR, 200 independent simulation are carried out. In Figure 3(a), one source is fixed at 10°, the other source is located at 10° + Δ*θ*. vary from 0 to 30. In Figure 3(b), two sources are located at 10° and 20°. SNR vary from −5 to 20. It is shown from Figure 3 that JSDOA has the higher probability of resolution than MUSIC and L1-SVD.

### 4.4. Spatial Spectrum Comparison for Coherent Sources

**Figure 4.**
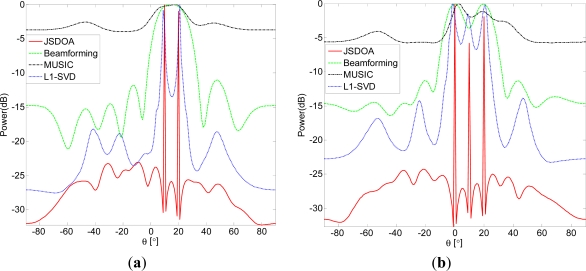
Spatial spectrum comparison for coherent sources. (**a**) Two coherent sources; and (**b**) Three coherent sources

In this simulation, we compare spatial spectrum for completely coherent sources. Set *SNR* = 10, *T* = 20, $\delta_{1}={\text{max}}_{i}\left|x_{i}^{0}\right|/\sqrt{2}$, *δ_j_* = *γδ_j_*_–1_, *γ* = 0.5, *J* = 7. In Figure 4(a) two coherent sources, located at 10° and 20°, are considered.

In Figure 4(b) three coherent sources, located at 10° and 20°, are considered. It is shown from Figure 4 that JSDOA and L1-SVD can be used to estimate DOA for coherent sources and that JSDOA has higher resolution than L1-SVD.

## 5. Conclusions

In this paper, a joint-sparse recovery algorithm, called JSDOA, is proposed to estimate DOA with sensor arrays. We pose the DOA estimation problem as a joint-sparse recovery problem, which can be solved by minimizing the approximate *L*_2,0_ norm. In particular, the *L*_2,0_ norm is approximated by an arctan function to represent spatial sparsity and the approximate *L*_2,0_ norm minimization problem is solved by a quasi-Newton method. Finally, the proposed algorithm is examined by simulations. Several advantages over existing DOA estimation algorithms were identified. It can perform well with a limited number of snapshots, while the number of sources need not be known *a priori*. Besides, it improves the resolution, and it can also handle the coherent sources well.

## Figures and Tables

**Table 1.** The main steps of JSDOA.

Algorithm 1: joint-sparse DOA estimation
Input: *A*, *y*
Initialization:
(1) Set *X*^0^ = *A^T^*(*AA^T^*)^−1^*Y*
(2) Select a decreasing sequence *δ* = [*δ*_1_*δ*_2_ ⋯ *δ_J_*], set *ɛ* and parameter *λ*
Iteration:
(1) for *j* = 1,2, … *J*
(2) solving (14) by BFGS algorithm
(2–1) *x* = *x*^j − 1^, *H* = *I* (*I* is unit matrix);
(2–2) while *norm* (∂*L_δ_j_,λ_* (*X*)/∂*X*) > *δ_j_ɛ*;
(2–3) Search step length $\tilde{t}:\tilde{t}=\underset{t}{\text{min}}L_{\delta_{j},\lambda }\left(X-tH\partial L_{\delta_{j},\lambda }(X)/\partial X\right)$
(2–4) Let *X* = *X* – *t̃H*∂*L_δ_j_,λ_* (*X*)/∂*X*
(2–5) Update the matrix by (16)
(2–6) end
(3) Let *X^j^* = *X*
(4) end
Output: *X̂* = *X^J^*, spatial spectrum *P*(*θ̄_i_*) = 10 log_10_ (‖*X̂*(*i*,:)‖_2_)

## Appendix—The Conjugate Gradient of *L_δ,λ_*(*X*)

From expression of *F_δ_*(*X*), we have:

(18) $$\frac{\partial F_{\delta }(X)}{\partial X_{R}}=\frac{1}{\delta^{2}}\Lambda X_{R}$$

(19) $$\frac{\partial F_{\delta }(X)}{\partial X_{I}}=\frac{1}{\delta^{2}}\Lambda X_{I}$$

where

$$\Lambda =\mathrm{diag}\left\{\delta^{2}/(\xi_{1}^{4}+4\delta^{4}),\delta^{2}/(\xi_{2}^{4}+4\delta^{4}),\cdots ,\delta^{2}/(\xi_{n}^{4}+4\delta^{4})\right\}$$

According the definition of conjugate gradient, we get:

(20) $$\frac{\partial F_{\delta }(X)}{\partial X^{*}}=\frac{1}{2\delta^{2}}\Lambda X$$

Set

$$G(X)={\left\|\Phi X-Y\right\|}_{F}^{2}=\mathrm{tr}\left[{\left(\Phi X-Y\right)}^{\prime }(\Phi X-Y)\right]$$

where ‖•‖*_F_* denotes Frobenious norm and *tr*[•] denotes the sum of diagonal entries. We have:

(21) $$\frac{\partial G(X)}{\partial X_{R}}=-2\;\text{Re}\left[\Phi^{\prime }(Y-\Phi X)\right]$$

(22) $$\frac{\partial G(X)}{\partial X_{I}}=-2\;\text{Im}\left[\Phi^{\prime }(Y-\Phi X)\right]$$

Then the conjugate gradient of *G*(*X*) can be written as:

(23) $$\frac{\partial G(X)}{\partial X^{*}}=\Phi^{\prime }(Y-\Phi X)$$

From (20) and (23), we have:

$$\frac{\partial L_{\delta ,\lambda }(X)}{\partial X^{*}}=\frac{\partial F_{\delta }(X)}{\partial X^{*}}+\frac{\partial G(X)}{\partial X^{*}}=\frac{1}{2\delta^{2}}\Lambda X+\lambda \Phi^{\prime }(Y-\Phi X)$$
